# Home away from home: international students' experiences during the COVID-19 pandemic and the role of US higher education

**DOI:** 10.3389/fpsyg.2023.1104200

**Published:** 2023-09-12

**Authors:** Xiaoqiao Zhang, Kelly C. Hsu, Kirsten E. Fleming, Cindy H. Liu, Hyeouk Chris Hahm

**Affiliations:** ^1^School of Education, Shanghai Jiao Tong University, Shanghai, China; ^2^School of Arts and Sciences, Tufts University, Medford, MA, United States; ^3^College of Health and Rehabilitation Sciences: Sargent College, Boston University, Boston, MA, United States; ^4^Department of Pediatrics, Brigham and Women's Hospital, Boston, MA, United States; ^5^Department of Psychiatry, Brigham and Women's Hospital, Boston, MA, United States; ^6^Harvard Medical School, Boston, MA, United States; ^7^School of Social Work, Boston University, Boston, MA, United States

**Keywords:** COVID-19 pandemic, international students, US higher education, role of university, qualitative study

## Abstract

**Introduction:**

This study focuses on the impact of the COVID-19 pandemic on international students' overall experiences.

**Method:**

We interviewed 22 international students from 11 countries and 17 universities in the US who participated in a large longitudinal study that aims to understand the physical and emotional wellbeing of young adults during the COVID-19 pandemic. Guided by Bronfenbrenner's ecological model, the findings suggested that students were impacted by the COVID-19 pandemic at interpersonal, institutional, political, and personal levels.

**Results:**

The results showed that the pandemic exacerbated existing stressors such as the lack of social support from family, various visa regulations, competitive and limited job opportunities, discrimination and xenophobia, particularly toward students from Asia, and financial burdens. Additionally, the findings highlighted students' perceived loss of the “American dream” and the uneven return on investment due to the pandemic.

**Discussion:**

This study reveals the importance of US higher education institutions in supporting international students during the pandemic, particularly in terms of their sense of belonging. Recommendations for institutions drawn from the findings are proposed to better support international students during times of COVID-19 and beyond.

## Introduction

In 2019, there were over one million international students in the United States (Institute of International Education, 2020). In total, international students have contributed $44.75 billion to the U.S. economy (Institute of International Education, 2018). Aside from their financial contribution, international students play an essential role in diversifying university campuses and their host communities (Leong, 2015).

There are many reasons why students choose the United States as a destination for study. They may gain potential stature from these universities and receive economic benefits after earning their degree (Chen et al., 2015). By studying in the US, students can broaden their experiences and gain skills in critical thinking (Shostya, 2015). Despite the many positive outcomes of these experiences, international students face numerous challenges and stressors including language barriers, sociocultural differences, racism and discrimination, and financial and immigration concerns (Smith and Khawaja, 2011).

The COVID-19 pandemic is likely to exacerbate international students' existing stressors, such as visa regulations, travel restrictions, time zone differences for online classes, and financial burdens (Eidt, 2020). International students being one of the vulnerable groups during the pandemic, finding both physical and mental supports were crucial. During the initial outbreak of the COVID-19 pandemic in the US, a university president stated in his outreach email to his international students that he understood their hardships and reassured them that the university is their “home away from home”. His one simple message that the university cared tremendously impacted the students.

The purpose of this study is to shed light on the real-life experiences of international students during the COVID-19 pandemic and to uncover factors that influence their decisions and everyday experiences. Such insights can inform host communities and educators to better support international students during and after the COVID-19 pandemic.

Much of the literature has focused on how international students are affected by various stressors, and how they develop certain coping mechanisms in adapting to a different host country (Yeh and Inose, 2003; McLachlan and Justice, 2009; Yan and Berliner, 2013; Wu et al., 2015). These stressors may come from educational and socio-cultural differences, administrative policies, and perceived and actual prejudice and discrimination (Yeh and Inose, 2003; Sue et al., 2007; Hendrickson et al., 2011; Yan and Berliner, 2013).

Navigating language barriers has been a major stressor for international students (Andrade, 2006; Gebhard, 2012). English fluency has a direct impact on students' confidence level, acculturation, academic performance and achievement, and their overall adjustment to establishing relationships with domestic students (Yeh and Inose, 2003; Sümer et al., 2008; Kuo, 2011; Hamamura and Laird, 2014). In terms of coping, international students are more likely to seek support from their peers and less likely to seek professional help (Heggins and Jackson, 2003). Having more host friendship networks is associated with decreased social isolation and greater satisfaction with their U.S. study abroad experience (Hendrickson et al., 2011).

As the COVID-19 pandemic perpetuates xenophobic attitudes toward Asians, particularly those of East Asian descent, it is important to recognize the complex and persistent history of discrimination and racial prejudice that have acted as stressors prior to the pandemic. Asian international students often face direct and indirect forms of racial prejudice and discrimination that negatively affect their mental health (Sue et al., 2007; Kim and Kim, 2010; Ong et al., 2013). Although there are instances of explicit acts of racism against Asian international students, much of the contemporary literature acknowledges a more subtle, disguised, and unintentional nature of discrimination (Sue et al., 2007). These covert forms of racial prejudice and discrimination or microaggressions are embedded in everyday experiences for individuals of Asian descent (Sue, 2010). Regardless of the original intention, targeted populations often feel devalued and stereotyped. As a result of societal normalization and the frequency of microaggressions, a growing number of studies have shown their damaging effects on mental wellbeing for Asians and Asian Americans, including disruptions to sleeping patterns as indications of psychological distress (Sue et al., 2007; Kim and Kim, 2010; Sue, 2010; Ong et al., 2013). On top of psychological distress, Asian international students may also distinctively experience and feel as if these acts are justified, as “foreigners” to the country (Yeo et al., 2019).

Despite these various challenges and stressors, international students continue to seek opportunities to stay in their host country. According to the “push-pull” model (Mazzarol and Soutar, 2002), one of the major motivators for international students to study abroad is the act of migrating into the country. The number of international students on Optional Practical Training (OPT) status, a temporary employment period for international students to seek opportunities within their field of study, doubled from 76,031 to 223,539 individuals (Institute of International Education, 2020) in the last decade prior to the COVID-19 pandemic. However, following the outbreak of COVID-19, various announcements were made which caused international students to feel insecure about their status. In particular, the U.S. Immigration and Customs Enforcement (ICE) announced in June 2020 that international students whose schools did not offer in-person courses could not stay or return to the United States (Metcalfe, 2020). Despite the policy being overturned the same week, it nonetheless created uncertainties about immigration policy, leading to greater stress for international students. These uncertainties, compounded with unanticipated transitions and increased racism, may have further affected Asian international students' mental overall experience and wellbeing.

### Conceptual framework: bronfenbrenner's bioecological theory of development

**Figure 1:**
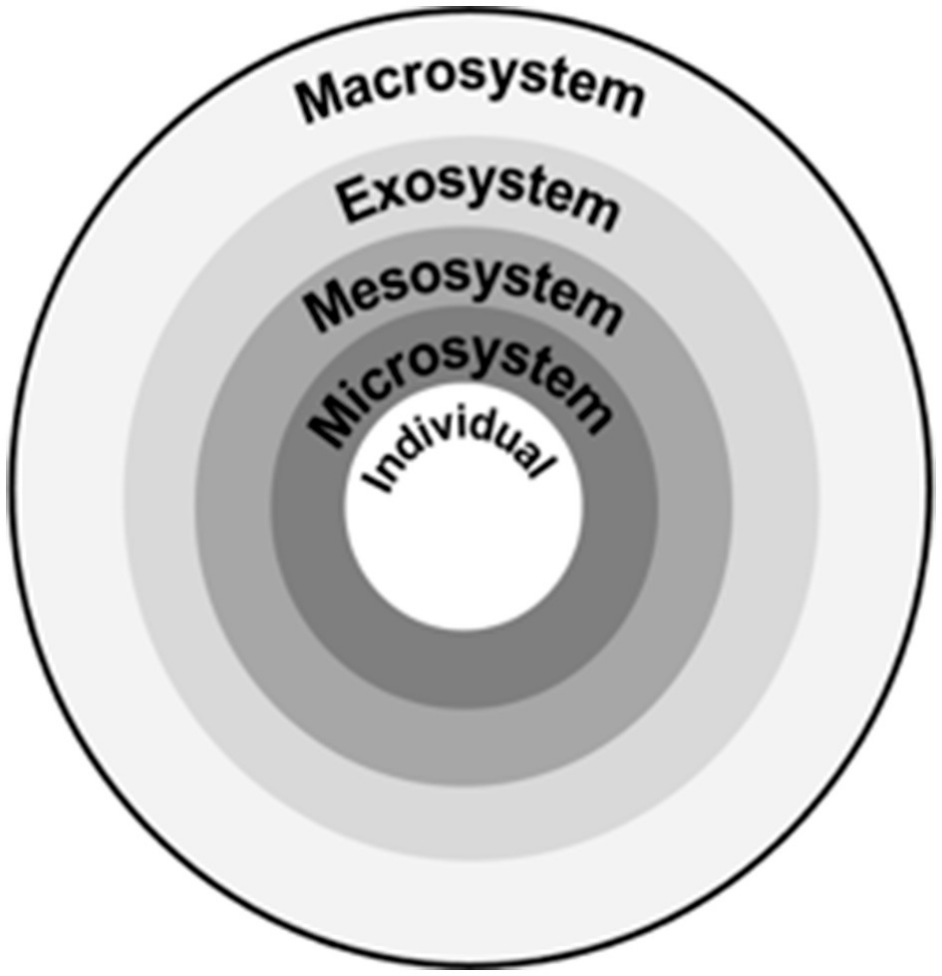
Adapted from Bronfenbrenner (1979, 1989, 2005) framework.

This study uses Bronfenbrenner's bioecological theory of development (Figure 1) to guide the study and explore international students' experiences through each level of their environment during the COVID-19 pandemic. The framework not only allows researchers to understand the experiences but also yields insights related to participants' interpersonal relationships, relationship to their institution, the local communities, relationship to the political system, and the impact that these have on international students.

Bronfenbrenner's model is divided into four main categories: the microsystem, the mesosystem, the exosystem, and the macrosystem. The four categories in Bronfenbrenner's theory interconnect and jointly affect the individual. Consequently, through these interchanges within their surroundings, the individual undergoes the formation of meanings and experiences (Bronfenbrenner, 1979, 1989, 2005).

The microsystem is the environment the individual is directly engaged in. This can include family members or friends. In the specific case of a university student, the microsystem consists of roommates, classes, and clubs or organizations the student belongs. The mesosystem is the interactions that take place between members of different microsystems, and how these systems interact with each other (Bronfenbrenner, 1977). For example, the interactions of students with their academic advisors, faculty, staff, friends, and parents, and the collection of their interactions form the mesosystem. The mesosystem is largely relationship-based and focuses on the connections between different microsystems. Recent studies show that the interactions between these systems are important for forming students' support networks and play an important role in impacting their experiences (Zhang, 2018). The exosystem is again even further removed from the individual but still plays an important role in impacting them. The exosystem includes entities or organizations the individual may not access themselves, but still influence the individual by encompassing or affecting the settings where they are found (Bronfenbrenner, 1977). For an international student, this can include U.S. foreign policies, immigration laws, visa regulations, and academic requirements for international students (Zhang, 2018). The more removed from the individual that these levels are, the more they encompass. The macrosystem, the final level of Bronfenbrenner's theory, includes customs, politics, and views of the society that the individual belongs to. In the macrosystem, cultural values are also included. This level of influence, although it is the farthest removed from the student, can still include important structures that impact an international student studying in the United States. These include the culture surrounding higher education in the US, expectations for college, perceptions of gender roles, occupation, and also cultural and language differences (Zhang, 2018). All of these levels have an impact on the individual's functioning within society.

Bronfenbrenner's model will serve as scaffolding to understand the specific impacts of the COVID-19 pandemic on international students' lives. Bronfenbrenner's micro-, meso-, exo-, and macro-systems provide insight into how relationships influence psychological development.

## Method

An exploratory case study employed a qualitative research method in this study. Due to the temporal proximity to the onset of the COVID-19 pandemic and inclusion criteria, the participant pool available for this study was limited. Nevertheless, a case study can offer in-depth insights into a complex phenomenon or individual experiences. The objective of this research was to explore and yield insights into the lived experiences of international students during the beginning outbreak of the COVID-19 pandemic. A case study is used to answer research questions on “how” and “why” the method approach normally focuses on contemporary events and minimum manipulation of the data (Yin, 2014). This is an ideal approach to determine how the COVID-19 pandemic has impacted international students.

### Data collection

Data were drawn from a larger project, which focused on understanding the physical and emotional wellbeing of young adults during the COVID-19 pandemic. Recruitment for the study took place through their university student listservs departments' listserv announcements and through social media (e.g., Facebook). Among the survey respondents who participated in the first wave of data collection (*n* = 1,223), 57 participants self-identified themselves as international students. For students to be recognized as international students, they must meet the following criteria: (1) those who did not have US citizenship; and (2) those who were enrolled in a US institution of higher education. After four rounds of recruitment, 35 (61%) participants agreed to have a follow-up interview. All participation was voluntary. All participants received a $20 Amazon gift card following the interview.

The interviews were one-on-one, semi-structured, and conducted over HIPAA-compliant Zoom accounts. The recordings of the interviews were then stored in the research team's secured shared drive. Each interview took 45 min to 1 h to complete and was conducted in English. Interviews were primarily conducted by the first author and by trained student research assistants.

### Participants

Among the 35 participants who agreed to in-depth interviews, two participants' data were lost due to the student interviewer's technical difficulties, while others did not respond to the follow-up recruitment email. Out of the recorded interviews, 22 interviews were successfully transcribed and used for analysis. Of the participants, there were 20 female and 2 male students. Countries represented were Argentina, Canada, China, Hong Kong, India, Japan, Lebanon, Malaysia, Oman, Pakistan, South Korea, and Vietnam.

Table 1 presents participants' country of origin, gender, level of education, type of universities they enrolled in, and the states where institutions were located.

**Table 1:** Participant details.

**Country of origin**	**Gender**	**Student status**	**Type of university**	**State of university**
China	M	Graduate	Small private research institution	MA
Japan	F	Undergraduate	Large private research institution	MA
South Korea	F	Graduate	Large private research institution	MA
South Korea	F	Graduate	Large private research institution	CT
Argentina	F	Undergraduate	Large private research institution	MA
China	F	Graduate	Liberal arts college	MA
Hong Kong	F	Graduate	Large public research institution	PA
China	F	Graduate	Large public research institution	VA
China	F	Undergraduate	Large private institution	MA
Canada	F	Undergraduate	Liberal arts college	MA
India	F	Undergraduate	Large private institution	GA
China	F	Graduate	Large private institution	WA
India	F	Undergraduate	Large private institution	MA
India	F	Graduate	Large public research institution	GA
India	F	Undergraduate	Large private research institution	MO
Pakistan	M	Undergraduate	Liberal arts college	SD
Lebanon	F	Graduate	Large public research institution	ND
India	F	Undergraduate	Large public research institution	WY
India	M	Graduate	Large public research institution	WY
South Korea	F	Graduate	Large private research institution	IL
Malaysia	F	Undergraduate	Large public research institution	MI
Vietnam	F	Graduate	Small private research institution	MA

Thematic analysis (Braun and Clarke, 2006) was chosen as the main tool for analyzing all of the data. With the analysis approach, we anticipated to explore and present the participants' experience fully by immersing ourselves in the data while using their authentic experiences to shed light on any possible underlying stressors. Open coding was performed by two authors (XZ and KH) for the first two rounds of analysis.

The conclusions and outcomes of this process were derived through open coding guided by theoretical propositions. In order to maintain credibility in the findings, as emphasized by Creswell (2012), the first author transcribed and coded all 22 transcriptions. The second author coded two interviews which she conducted and transcribed six others. After the first and second authors reached a consensus on the initial codes and emerging themes from the same two interviews (Braun and Clarke, 2006), the first author identified and developed common themes across all participants' interviews. To ensure the proper interpretation of data and findings, the first author conducted member checking with the second author through 3 months of weekly meetings, discussions with the other authors, naming themes, and write-ups.

## Results

**Figure 2:**
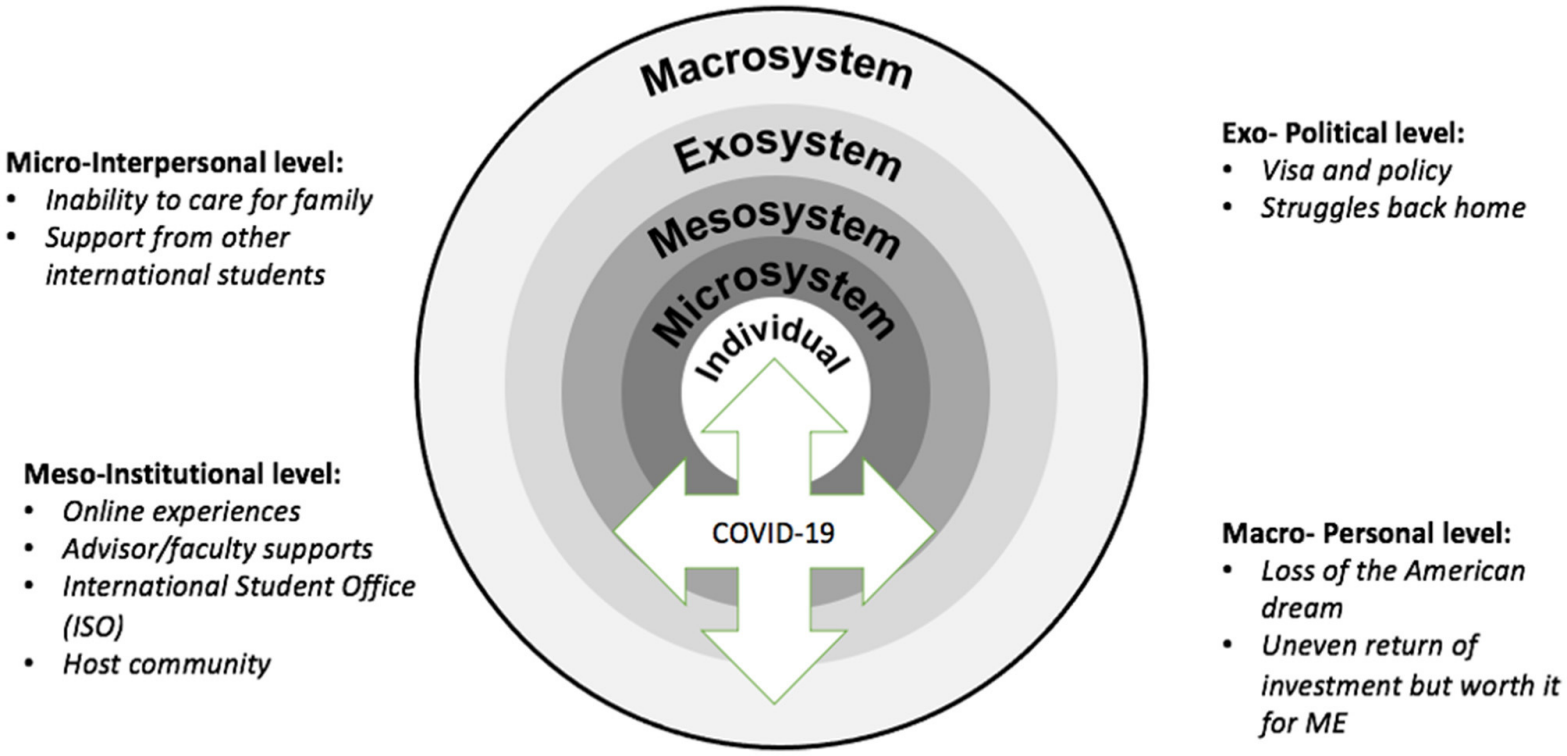
Application of Bronfenbrenner (1979, 1989, 2005) framework in understanding international students' experiences during COVID-19.

After rounds of analysis guided by Bronfenbrenner's ecological framework (see Figure 2), we categorized these themes into four different levels and demonstrated how the COVID-19 pandemic has impacted international student's experiences during the beginning stage: interpersonal level, institutional level, political level, and individual level. Each theme within the level has impacted the participants simultaneously and interactively.

### Interpersonal level (microsystem)

#### Inability to care for family

For participants who were from Asia, their worries about the pandemic began in January. Many were stressed for their family members, especially those who were older. As the case numbers began to increase in the US, families back home, in turn, also began to worry for the participants. One participant's father got affected and lost his job, but the hospital in her country was not equipped to handle the case. In the end, he had to fly to a nearby country to be treated.

He was so traumatized. And the problem for me is that [pause] I couldn't just take an airplane, go see him because if someone is infected that you cannot, there was no way that I could go see him... It, was really hard to being alone in a studio, like in a room [pause] knowing that your father is like in very critical conditions.

#### Support from other international students

In response to the challenges stemming from COVID-19, many participants described friends—including roommates—and significant others. The more support systems in place for students, the greater levels of coping mechanisms and lower feelings of isolation for these participants. Despite the support from domestic students, some participants instead sought emotional support, guidance, and updates from other international students they knew, despite not being in the same institution.

I had a few international friends already in the US, who are like, in different stages, like either like applying for the H1B, or still at their OPT, ‘um and so we all connected it and that was really good too. ‘um, and I think it was disappointing because of so much of the process was just international students talking to other students, and I didn't really feel that I was getting good guidance from the school or even like an assurance that they cared about us. [sic]

### Institution level (mesosystem)

#### Online experiences

Since March, classes and graduations have been online. Graduates expressed their disappointment that they were unable to walk across the stage and get photos with friends and family. Despite institutions promising possible make-up ceremonies, participants believed that the experience would never be the same. Additionally, the majority of the participants are currently still living in the US as a result of anticipating challenges such as time differences, internet instability, distractions from families, and the fear of not performing well academically if they were to leave. Frustration with the online experience was common as many lost out on in-person interactions, and perceived online classes as an indication of poorer academic quality. Some had difficulty with English since they relied on classroom interactions to understand and some missed out on hands-on experiences. As one nursing major participant put it,

like you know, I was in the maternity rotation. I was supposed to be learning how to catch a baby. And, I had to learn that online, [laughing but feeling frustrated], and my feeling was like, oh so I went to 6 h of Zoom class on catching a baby so now I can just do it? Like I was, I felt like got cheated of my education, and then I was like this is, like nursing is all about the hands on. I've gotta smell things, and like see things, and hold people's hands, and so that's really disappointing. [sic]

#### Advisor/faculty supports

Despite many unexpected changes and disappointments, some international students' experiences were made positive through the support of their advisors or faculty members. In response to potential academic struggles, faculties provided flexible grading (i.e., pass/fail) to make the experience more manageable. In response to emotional struggles, faculties regularly checked in with participants and offered encouraging words through emails. It was clear that these experiences improved participants' mental wellbeing and increased their sense of belonging. For one Chinese participant, a hug and warm welcome from a professor greatly alleviated her fear of potential discrimination upon her return from winter break:

I do feel very supported. And my Actually, I feel a very strong sense of belonging here. That's why- that's also why I kept I stayed here for my PhD instead of going other universities. Um...first, my professors they're very caring. And yeah, they also check in see- yeah, they were still asking me whether I feel very isolated or whether I have people to talk to, whether we are organizing activities yeah and -and the uh program- the directors, they also- they also send out emails and actually remember in March...or April, I received an email from the director, I was very touched by the email. [sic]

### International student office

The International Student Office/Affairs had an immense impact—both negative and positive—on participants' overall transition during the pandemic. The office played a role in providing students' support, especially as a place for security and comfort. When the ICE policy targeting international students was initially announced, ISOs responded quickly and clearly made students feel better, despite the uncertainty surrounding their visa statuses. The ISOs that went a step further by personally and publicly advocating for students provided even more stability for students. One participant reflected:

Like our university, or like the International Programs Office told us that “no matter what the case is, we [the university] will do our best to like keep you here. And not like if you had to, like offer an in-person class for you guys alone, we will do that. We will do like everything we could!” So like, you know, like all these things that they might seem like little things, but they mean so much to you. Coming from them like, make this, just be sure that they are people that support you. Not only your friends and family or relatives but also like the International Programs Office.

However, participants who experienced the opposite felt greater stress and frustration. Examples include delayed responses, unclear information, confusing runarounds with other offices, and short-staffed offices leading to one person handling everyone's cases.

### Host community

The local ethical/faith communities and families reached out to participants through social media to offer additional support when COVID-19 hit. Despite not knowing these students, families within the community would offer their homes for students to stay in and garages for them to store their belongings in. Some families even helped on-campus students move and contributed to emergency relief funds on social media. Participants who had these supports had a more positive overall attitude toward the community and the country. On the other hand, the lack of support from and negative interactions within the local community generated a negative outlook for participants. Negative interactions were linked to concerns over mask-wearing, especially for Asian international students who feared discrimination from local community members:

Back then I think like within when I, as long as I reached a building I don't wear mask anymore so... hm, I mean that doesn't sound good now but, but... yeah, that's just what happened back then. So I didn't like, but then my lab everything was fine. When I go out I do have some weird stare but... honestly, I wasn't... physically or mentally harmed.

### Political level (exosystem)

#### Visa and policy

Prior to COVID-19, international students already faced paperwork and interviews with visa applications as well as limited job and internship opportunities. However, none of these worries were as concerning when compared to the ICE policy announcement in July. In response to the policy, participants described feeling fearful, anxious, concerned, panicked, angry, frustrated, disgusted, sad, uncertain, and even laughable. Fortunately, much like the participants had predicted during that time, the policy was overturned within a week. However, many felt that introducing the policy at all was unnecessary, exacerbating the already stressful visa status process. Already vulnerable to unexpected changes and transitions, international students did not need to worry about their ability to remain in the country as a student.

Yeah yeah, so when they reverse the policy, [laughing], I didn't feel better that I thought I would. I didn't feel like I'm safe now. I still felt completely unsafe, uncertain, and really afraid of ICE. I, I, I think I was really paranoid in that in those few weeks, like I remember I was running research project where we had to record ourselves, and I like backed out last minute like right before, because I was like I don't feel safe being recorded but I don't know who's gonna have to information, and I don't want people to know that I am an international student because I just felt so vigilant and like people were out to get me. And I also felt like well if they're just going to reverse it, then why did they even do it in the first place? Like what's the point? Like if they didn't need to, if this wasn't a necessary step for them, and they were just going to reverse it before it even went to court? Then like, it was clearly not worth much and so why would they put students through all this emotional turmoil just to be like, no, never mind! And I was furious, I was so upset that they would just play with our lives like that. [sic]

#### Struggles back home

Aside from struggling to keep up with the changing policies of the US, some participants were also dealing with problems back home. Some concerns included limited medical resources, the closed border policy, strict travel bans, limitations with the internet, and limited space within their homes. On top of that, one participant from Lebanon shared her experience after the explosion occurred in Beirut on 4 August 2020:

Um, well one thing that is just specific to my case, what happened in Lebanon about a week and a half ago was a major explosion that really just stressed, stressed us out a lot. And to top it all off, I think that was just a very hard week for me and every Lebanese really. And being abroad during that time just, just amplifies the difficulty. And so, you know, not only are you dealing with the start of internship with making sure dissertations over but you're dealing with just the trauma of seeing all these people dying and not, not being sure of, again, what you know, the safety of your loved ones and all that. So I think this you know, it's just this is just the one added thing that I think many people don't understand about a lot of just the instability in a lot of countries. [sic]

### Personal level (macrosystem)

#### Loss of the American dream

Perhaps the most frustrating result of this pandemic was the perceived loss of the American dream. When asked about their original motivations to come to the US, many participants recounted their idealization of the US as a representation of the land of opportunities, freedom, independence, and great diversity and inclusion within the country. Yet, after experiencing the disadvantageous policies toward international students and witnessing the events following the death of George Floyd, some participants had begun to reconsider their plans to stay to work or eventually apply for citizenship.

I think that this year, like the things that have happened to international students are really disillusioning me, but it's also all the ways that the country treats its own people that are really like, disappointing. Like just the way that they, they are so horrible to their own citizens. It makes me think like, okay, what's the end goal here? So I become a citizen? I still get treated the same way. You know? Like I may not face the same bureaucratic challenges, you know, of like having to renew my visa or whatever, but the country hasn't shown me that like, Oh, once you become a citizen, we will protect you. So why am I still here, you know?

#### Uneven return on investment but worth it for ME

Many participants believed and accepted the idea that schools admitted international students mainly as a financial contribution to the institution. However, in light of recent events, participants felt that the investment they made both financially and personally had turned into a burden instead. After years of contributing and making progress in the country, international students felt that they had to pack up and leave everything they have built behind. As a result, it was impossible to return the investment they and their family initially made.

Despite the challenges participants had and continue to face, all participants believed that overall, their decision to study in the US was worth it for their personal growth and the overall experience. Participants stated many different examples for this: the opportunity to live independently, to engage in a diverse student and faculty population, and to find and gain skills in new research opportunities and equipment. However, in terms of financial investment, no participant believed it was an even return based on the actual cost and emotional turmoil of their journey. As a student who received a full scholarship from the university reflected,

Um, I, I'm very happy with the education I got and the experience I had, but it's, I don't know if it's something I would necessarily recommend for other people....Um just because...I think [University]'s tuition is $70,000 a year. And when you think about the experience and the education you have, maybe the job prospects that are available, I don't know if it's really worth it. Regardless of whether international students can...can afford this education. Um, yeah, I don't- I don't really know if it's worth that much. [sic]

## Discussion and implications

This study has explored international students' experiences during the beginning of the outbreak of the COVID-19 pandemic. Many experiences participants reflected upon in this study were not new for international students, such as encountering hardships of being far away from loved ones, navigating challenges in finding internship and job opportunities, and looking for strong social support systems with friends or significant others. Due to the COVID-19 pandemic, particularly for Asian international students, the “double trauma” of having already witnessed loved ones in Asia combating the initial wave of the epidemic and experiencing the sudden rise of global racism puts them in a more vulnerable position. Throughout the entire study, one prominent theme emerged: the university in the international students was enrolled and their overall attitudes, along with other factors, such as fellow students, faculty, administrators, policies, and the host communities, all played a significant role in shaping the experiences of these international students during this pandemic.

### Microsystem

When participants were extremely vulnerable to being away from their homes, the university played an essential role. The participant whose father got affected by COVID-19 responded that the only thing she wished for was to have the university staff “listen”. She reflected that when seeking support from various offices, due to the lack of patience, limited knowledge of the international students' status, and short-staffing, all others could do was send her to the international student office. Yet, some unique needs, such as financial aid issues, may not belong to the international student office within that institution. Most participants responded that more professionals should be hired with the financial contribution they make toward the institutions, and more effort should be put in place to support them. It is advisable for institutions to contemplate the creation of specialized financial aid programs aimed at addressing the distinct situations of international students. With the suggestions from the participants, host institutions can establish clear and transparent communication channels (e.g., all related office administrators and international student ambassadors) to provide guidance and resources for international students to navigate available supporting options.

### Mesosystem

Universities have been seeking ways to support and engage students to help them transition into the new culture. As studies have shown in the past, the interactions between international students and their faculty, domestic students, and the host community as a whole have a positive impact on international students' sense of belonging and overall life satisfaction (Glass et al., 2015; Le et al., 2016; Zhou and Cole, 2016). This study supports this argument. Despite the challenging experiences participants have gone through, particularly during the pandemic, many of the participants shared that the greater the sense of belonging they felt with their host university community or community individuals, the more tolerable the overall experience was. Through our findings, we also learned that in addition to faculty and domestic students, interactions with individual institution members, as well as the institution, as a whole's attitude and actions to support participants during the pandemic impacted their sense of belonging.

### Exosystem

Particularly when the ICE policy was announced, there was/it was a shocking wake-up call to these students as they realized that being an F-1 student did not fully secure their status in the US. However, as many participants indicated, their university was still their safety nest. Most of them were not surprised when Harvard and MIT joined together to overturn the proposal, despite not studying at that institution. Though, some still wished their universities would step up earlier and communicate with them more often. Clear guidelines and information, particularly regarding their visas and ways to support international students was something they wish universities could further develop for future international students.

### Macrosystem

Finally, despite all of the challenges students are facing, they still wish to have an opportunity to stay and gain working experience even with the intention not to immigrate. However, what we learned from this study was the stress of post-graduation. Many participants have made financial and emotional contributions to their new country and culture. However, the pandemic made them realize their reality of being a temporary sojourn in the host culture. Many participants suggested that they will not encourage their friends or family to come to the US in the future but instead go to other countries where they are welcome to stay.

Even though the Institute of International Education' press release shortly after the pandemic indicated, due to the impact of COVID-19 in 2020, universities are anticipating a drop of more than 40% in the enrollment of international students in the upcoming year (Institute of International Education, 2020). Yet, the overall number of internationals only dropped within a short period of time. However, more students begin to explore more options besides the U.S. to study after the fact. To continue the effort in recruiting international students, it is important to seek ways to increase the effort to smoothen the overall experience while establishing policies and ways to better support students' future whether in the US or elsewhere.

With the findings indicated in the study, Table 2 lists recommendations for institutions to seek ways to support their international students and the greater community.

**Table 2:** Recommendations to better support international students.

**Recommendation**	**Anticipated outcome**
*Increase capacity:* Maintain a healthy ratio of international student office officers and students	Better personable interaction and a manageable workload
*Increase understanding:* Educational workshop on basic knowledge of international students for all faculty and staff	A better understanding of supporting and advising international students
*Increase communications:* Create shared experience outside the classroom with domestic/international student leaders	Provide space and opportunities for students' exchange and engagement
*Increase engagement from the local community:* Create a resource list from the local community	Better communications within the greater community
*Increase cross-cultural knowledge:* Training/workshop on cross-cultural counseling	Better understanding and communication flow between students and institution members
*Increase opportunity:* Partnership with alumni and organizations outside of the US	Provide internship opportunities for international students post-graduation despite not being in the US
*Increase advocacy:* Advocate and be the voice for international students in bettering the future international education policies	Efficient visa process; Effective policies for more possible post-graduate working opportunities.

## Limitations

Several limitations should be noted about the participants and sample data. Out of 22 participants, only 3 were male students. It was visible through transcription that in terms of the overall tone of the interviews regardless of the xenophobic incidents, uncertainties with visa statuses, limited friends around, and the loss of normalcy in life, the male participants responded much more calmly than female participants. It will be worth exploring further why there was a female majority in the participation of such surveys and studies and if gender played a role in experiences during the COVID-19 pandemic.

This study was able to recruit students from 17 different institutions. Depending on the size, location, and overall number of international students of the institution, it was evident that the attitude and support levels were different for smaller liberal art colleges with fewer international students. These colleges had more capacity to accommodate various changes including the ICE policy. It will be worth exploring whether a university's size, location, and the number of overall international students played a role in international students' experience.

## Conclusion

This study was conducted within months of the COVID-19 pandemic, with a specific focus on the experiences of international students. It was observed that international students, similar to other non-international participants in the large study, faced numerous negative phycological challenges including anxiety, PTSD, and lack of sleep (Liu et al., 2020; Hyun et al., 2021). However, this study revealed several unique challenges that were specific to international students, such as being away from family, dealing with visa regulation restrictions, and coping with time zone differences. Notably, the study underscored the crucial role and attitude of the host institution in fostering a sense of belonging and promoting better mental health among international students during times of crisis.

As universities and programs continue seeking ways to engage and increase international student enrollment on campus, it is also important to create better experiences and offer increased support. This study suggests that the more support students had from their institutions, university staff members, faculty, and host communities, the more tolerable the COVID-19 pandemic was for them.

In a letter addressed to all international students on his campus following the realization of the ICE policy, a U.S. university president expressed that the university serves as their “home away from home.” This study underscores the crucial role that institutions play in supporting international students and emphasizes the significance of collaborative efforts across all systems both during and beyond the pandemic.

## Data availability statement

The original contributions presented in the study are included in the article/supplementary material, further inquiries can be directed to the corresponding author.

## Ethics statement

The project was approved from Boston University—Charles River Campus IRB (Protocol #5541X). All investigators are certified in the responsible conduct of research involving human subjects. Written informed consent for participation was not required for this study in accordance with the national legislation and the institutional requirements.

## Author contributions

XZ: Conceptualization, methodology, data collection, data transcription, formal analysis, investigation, writing—original draft, writing—review and editing, and project administration. KH: Partial data collection, transcription and analysis, writing—original draft, and writing—review and editing. KF: Partial data collection and transcription, writing—original draft, and writing—review and editing. CL and HH: Conceptualization, writing—review and editing, supervision, and funding acquisition.
